# Removal of intracaval and intracardiac leiomyoma in a 49-year-old woman

**DOI:** 10.1016/j.jvscit.2023.101239

**Published:** 2023-06-02

**Authors:** Armin Tabiei, John M. Stulak, Sebastian Cifuentes, Amanika Kumar, Randall R. DeMartino

**Affiliations:** a Division of Vascular and Endovascular Surgery, Mayo Clinic, Rochester, MN; b Department of Cardiovascular Surgery, Mayo Clinic, Rochester, MN; c Division of Gynecologic Surgery, Mayo Clinic, Rochester, MN

Intravascular leiomyoma extending to the inferior vena cava (IVC) and right heart is a rare condition that generally occurs in middle-age women.^1^ Treatment consists of surgical tumor removal in a single-stage or staged interventions. Deep hypothermic circulatory arrest is often required to remove the intracardiac and intracaval portions of the leiomyoma.^2^ Recurrence of the leiomyoma has been observed in one third of patients who undergo incomplete resection; however, complete resection leads to excellent results with no recurrence.^3^

We report the case of a 49-year-old woman with no significant medical history who presented to an outside hospital because of palpitations and an episode of brief syncope. Echocardiography demonstrated a large thrombus in the right atrium extending into the right ventricle. A computed tomography scan of the abdomen and pelvis was performed, which demonstrated a large pelvic mass measuring ≤20 cm that was contiguous with thrombus in the bilateral gonadal veins, bilateral renal veins, IVC, and right atrium. Concerns for an intravascular leiomyoma were raised, and the patient presented to our institution for further care. Following a preoperative multidisciplinary evaluation, the patient was scheduled to undergo resection of the tumor through a laparotomy and sternotomy approach. The surgical procedure used to completely resect the leiomyoma consisted of total abdominal hysterectomy and bilateral salpingo-oophorectomy, followed by tumor thrombus removal from the IVC and right heart with the patient under deep hypothermic circulatory arrest (Supplementary Video, online only). Bilateral internal iliac vein ligation was performed to prevent hematogenous spread through this route if the tumor were to recur. Histologic examination of the tumor thrombus confirmed intravenous leiomyomatosis. On postoperative day 5, the patient had developed a subsegmental pulmonary embolism and was treated with a heparin nomogram. She was discharged home on postoperative day 13 with a 6-month course of apixaban (Eliquis; Bristol-Myers Squibb). At her 6-month follow-up examination, the patient reported lower extremity numbness that was possibly attributable to a stretch injury during the operation. No other symptoms were reported, and computed tomography demonstrated no tumor recurrence. No further anticoagulation therapy was prescribed, and the patient was scheduled for yearly follow-up. The patient provided written informed consent for the report of her case details and imaging studies.

## Appendix (online only)

Supplementary Video (online only) Surgical procedure used to completely resect the leiomyoma and remove tumor thrombus from the inferior vena cava (IVC) and right heart. *CT,* Computed tomography; *POD,* postoperative day; *RA,* right atrium; *RV,* right ventricle.
